# Tyrosine Supplementation Rescues a Growth Defect in a Humanized *S. cerevisiae* Model of YARS1 Associated with CMT-DI

**DOI:** 10.3390/ijms27167481

**Published:** 2026-08-21

**Authors:** Nancy Sun, Tristan N. Samuels, Kyle Hoffman, Ridhwan Busari, Zain Nasir, Nicole Girard, Noah M. Reynolds, Ilka U. Heinemann

**Affiliations:** 1 Department of Biochemistry, The University of Western Ontario, London, ON N6A 3K7, Canada; 2 Bioinformatics Solutions Inc., Waterloo, ON N2L 3K8, Canada; 3 School of Integrated Science, Sustainability, and Public Health, University of Illinois Springfield, Springfield, IL 62703, USA; 4 Children’s Health Research Institute, London, ON N6C 2V5, Canada

**Keywords:** amino acid supplementation, aminoacyl-tRNA synthetase, ARS disease, transfer RNA

## Abstract

Dominant pathogenic mutations in tyrosyl-tRNA synthetase (YARS1) are associated with Charcot–Marie–Tooth disease (CMT), a progressive peripheral neuropathy for which no disease-modifying therapies currently exist. While recent advances in amino acid supplementation therapies suggest potential benefit for recessive aminoacyl-tRNA synthetase disorders, their applicability to dominant *YARS1*-associated neuropathies remains unclear. Here, we investigated the pathogenic mechanisms underlying the dominant *YARS1* variants G41R, D81I, and E196Q. Using biochemical and functional analyses, we identified increased structural rigidity for G41R and E196Q proteins, while D81I is more susceptible to tryptic digestion. Furthermore, expression of the YARS1 variants in a humanized yeast model produced a dominant negative growth defect that is exacerbated at elevated temperatures, supporting disruption of canonical YARS1 function as a contributor to disease pathogenesis. Notably, tyrosine supplementation significantly rescued the observed growth defects across variants. These findings demonstrate that impaired tyrosine utilization contributes to the pathogenic effects of dominant YARS1 variants and provide proof-of-concept evidence that tyrosine supplementation may represent a potential therapeutic strategy for patients with *YARS1*-associated CMT.

## 1. Introduction

Accurate protein synthesis depends on the accurate charging of transfer RNAs (tRNAs) with their cognate amino acids, a reaction catalyzed by the aminoacyl-tRNA synthetases (ARSs) [1]. These enzymes establish the genetic code by charging each of the 20 proteinogenic amino acids to their cognate tRNA [2]. This highly specific reaction thereby ensures translational fidelity during protein synthesis. ARSs are essential in all organisms and are among the most highly conserved components of the translational machinery. In addition to their canonical roles in translation, several ARSs have been implicated in diverse cellular processes, including signaling pathways and stress responses [3,4,5]. Given their central role in cellular physiology, perturbations in ARS function can have profound consequences for cellular processes and health.

Given the highly specific and essential nature of ARSs, it is not surprising to find that variants in these proteins are often pathogenic. In recent years, pathogenic alleles in multiple ARS genes have been linked to inherited neurological disorders, particularly peripheral neuropathies within the spectrum of Charcot–Marie–Tooth disease (CMT) [2,6]. CMT, one of the most prevalent hereditary peripheral neuropathies, with an incidence of approximately 1 in 2500, represents a heterogeneous group of disorders characterized by progressive degeneration of peripheral nerves, leading to muscle weakness and sensory loss. There are two major subtypes of CMT: CMT1, which is characterized by the degeneration of the myelin sheath, and CMT2, which is characterized by the degeneration of the nerve axon itself [7]. Notably, seven cytosolic ARS genes have been associated with dominant forms of CMT, suggesting that subtle alterations in translational machinery can produce highly tissue-specific disease phenotypes [2,6]. Despite increasing recognition of ARS-associated neuropathies, the molecular mechanisms by which disease-associated variants disrupt cellular function remain incompletely understood.

**Figure 1:**
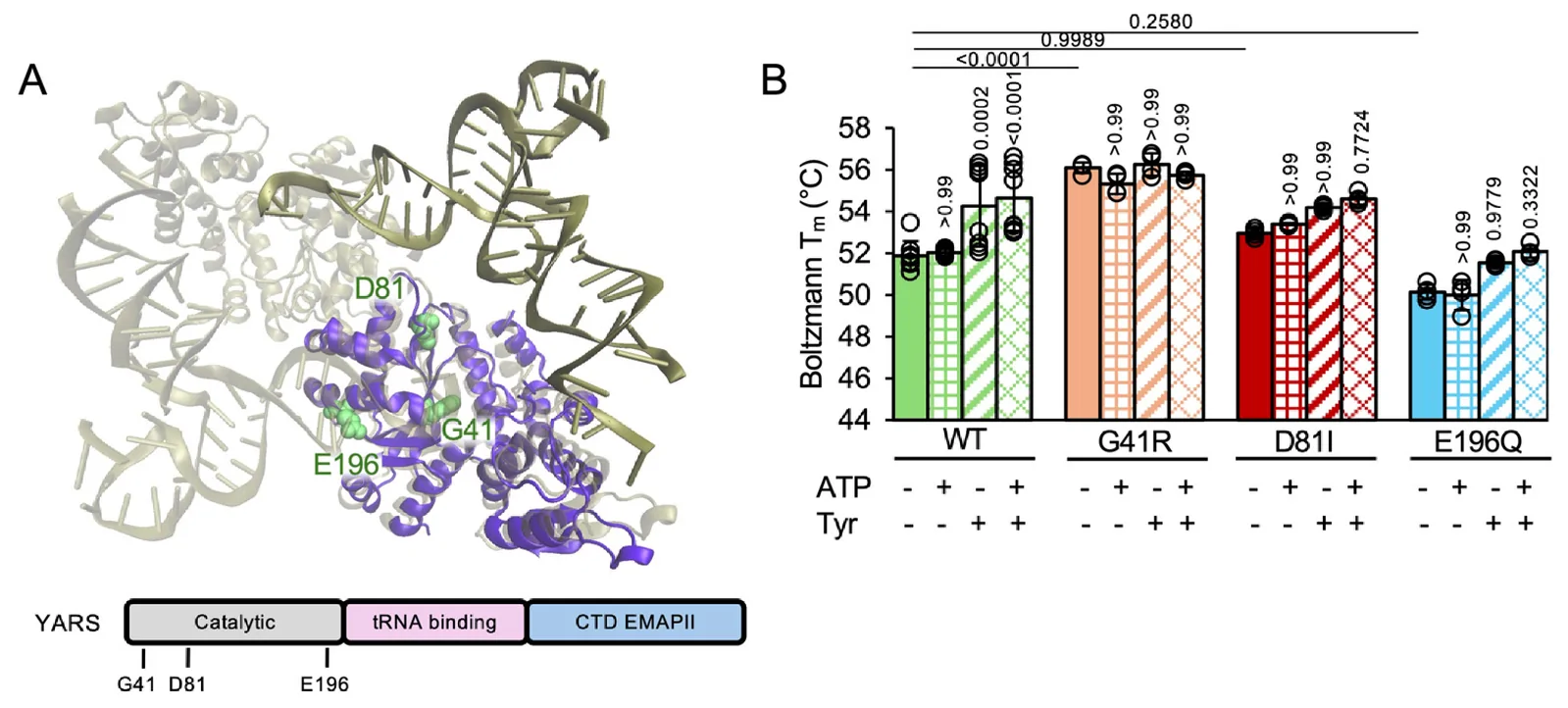

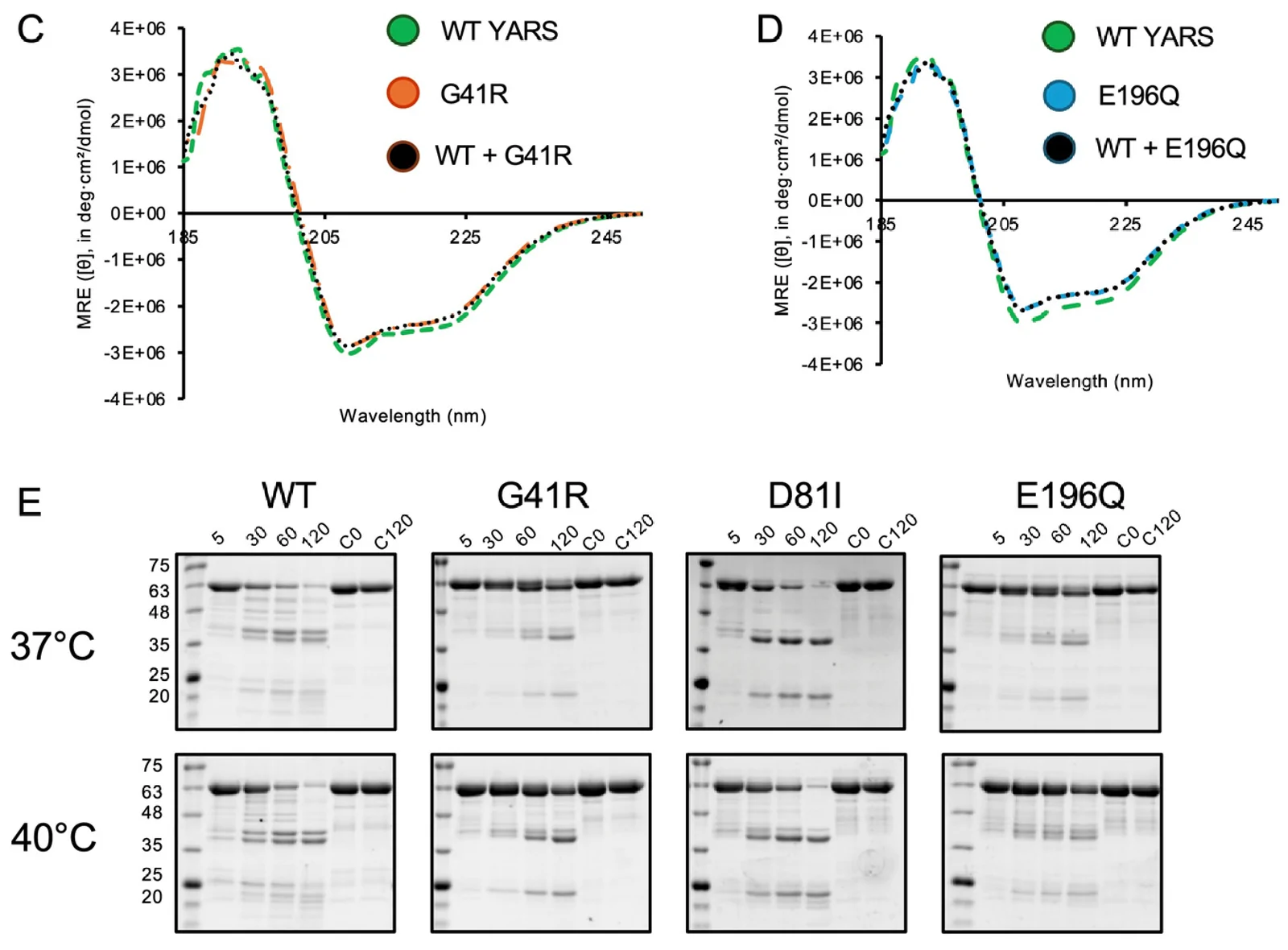
Protein biochemical characterization of recombinant CMTDIC-associated YARS1 variants. (**A**) The crystal structure of the yeast tyrosyl-tRNA synthetase in complex with tRNA^Tyr^ (tan), overlaid with the human YARS1 structure (PDB ID 4QBT, purple), visualized in VMD (PDB ID 2DLC). The G41, D81 and E196 residues are identified and colored green. YARS1 domain organization with variants analyzed here: (**B**) Melting temperatures of recombinant YARS1 WT, G41R, D81I and E196Q proteins. In sum, 1 μM of the proteins were incubated with or without 100 μM ATP or 200 μM tyrosine, or in a combination of 100 μM ATP and 200 μM tyrosine. Proteins were subjected to increasing temperature, from 25 °C to 95 °C, using the Protein Thermal Shift^TM^ Dye Kit and the QuantStudio 3 qPCR system. The melting temperature was approximated by fitting the Boltzmann equation, using the Protein Thermal Shift software (version 1.5.1). Bars indicate the mean ± standard error. (**C**) CD spectra of purified recombinant WT, G41R and (**D**) E196Q homodimeric or heterodimeric proteins, revealing a dominating alpha-helical secondary structure for all protein species. (**E**) Limited tryptic digestion of YARS1 proteins at 30 °C and 40 °C. Recombinant YARS1 WT, G41R, D81I and E196Q were equilibrated with buffer, then subjected to digestion by trypsin at 37 °C or 40 °C, with the time indicated in minutes. Control samples of YARS1 were incubated without trypsin for 0 or 120 min (C0 and C120). The reactions were quenched with SDS dye and boiling, then separated on a 12% denaturing SDS–polyacrylamide gel followed by staining with Coomassie blue to visualize the digest products. Statistics determined using one-way ANOVA in GraphPad Prism (version 10.3.1). *n* ≥ 3. Statistical comparisons to the WT are indicated directly above the error bars, which indicate one standard deviation.

One ARS implicated in dominant CMT is encoded by *YARS1*, which encodes the cytosolic tyrosyl-tRNA synthetase (YARS1). Some heterozygous variants in *YARS1* cause dominant intermediate Charcot–Marie–Tooth disease type C (CMTDIC), an axonal neuropathy characterized by progressive motor and sensory deficits, presenting with both demyelination and axonal degeneration [7]. Numerous missense variants have been identified in affected individuals, including G41R, D81I, and E196Q [8,9,10,11,12,13,14]. These variants are found in the N-terminal catalytic domain of the enzyme (Figure 1A), with the G41R variant having previously been shown to reduce aminoacylation activity, while a similar E196K variant retains catalytic competence in vitro [10]. Interestingly, however, both the G41R and E196K variants impaired motor performance and demonstrated electrophysiological evidence of neuronal dysfunction and defects in axon terminal morphology in a *Drosophila melanogaster* model of CMTDIC [10,15]. For other variants, including D81I, the biochemical and cellular consequences remain less well-characterized. These observations suggest that impaired aminoacylation alone may not fully explain the pathogenic effects of all *YARS1* variants and raise the possibility that additional cellular mechanisms contribute to disease.

In addition to the gap in the understanding of the molecular mechanism of CMTDIC, there is also no cure or widely recognized treatment strategy for the disease. Therapeutic approaches are largely restricted to symptom management through the use of physical and occupational therapy, as well as orthotics, braces and osteotomies to help correct skeletal deformities [16]. Recently, gene therapy and pharmacological strategies have been employed for specific CMT subtypes; however, they have shown variable success [16]. This lack of disease-specific treatment applies to the other six synthetases that are associated with dominant axonal CMT, as well as many other diseases that arise from variants in ARSs [2,6,17,18,19]. Given the understanding of the canonical function of YARS1 and other ARSs, it is presumed that increasing the availability of cognate substrates, such as the cognate amino acid, may help ameliorate symptoms by improving the overall activity of the enzyme. Increasing the amount of cognate amino acid may be sufficient to outcompete non-specific binding events with non-cognate substrate or increase activity to viable levels despite impaired enzyme kinetics or protein structure. Additionally, the non-canonical nuclear localization activity of YARS1 is related to cytosolic substrate availability, as only free YARS1 with the exposed nuclear localization sequence (NLS) can undergo nuclear localization [20,21]. As such, increasing the amount of cognate substrate may shift the equilibrium to favor the enzyme’s canonical activity in the cytoplasm. This hypothesis, that cognate amino acid supplementation may have a therapeutic effect for ARS-related diseases, has been supported in translational model systems [22,23] as well as in clinical trials [22,24]. Excitingly, in a recent longitudinal study examining the impact of a high protein and tyrosine-supplemented diet on biallelic *YARS1* patient outcomes, most patients demonstrated improved weight, language and motor skills, in addition to improvements in hematological parameters [25]. In vitro, patient fibroblasts showed significantly reduced aminoacylation activity, which was restored to near-control levels when supplemented with high levels of tyrosine [25]. Although promising, the responsiveness of ARS variants to amino acid supplementation is highly allele-specific [22], and has so far only been tested in recessive ARS diseases in the clinic. We seek to assess whether dominant, CMTDIC-associated *YARS1* variants will respond favorably to tyrosine supplementation. The budding yeast *Saccharomyces cerevisiae* has proven a powerful model for studying components of the translational apparatus because many essential translation factors are functionally conserved between yeast and humans [2]. In many cases, human ARS proteins can functionally complement their yeast orthologs, allowing the effects of human disease variants to be examined in a genetically tractable system. We have previously generated a humanized yeast model system for histidyl-tRNA synthetase (HARS1), which allowed us to characterize several CMT-causative *HARS1* alleles [22,23,26,27].

In this study, we established a humanized yeast model in which cellular growth depends on expression of human YARS1. Using this system, we examined the functional consequences of three CMTDIC-associated variants and found alterations in thermal stability and indications for structural changes in G41R, D81I, and E196Q. Expression of the disease-associated variants produced a dominant growth defect in yeast. Remarkably, supplementation with tyrosine significantly improved the growth of the mutant strains, suggesting that amino acid availability can modulate the cellular consequences of YARS1 dysfunction. Together, these findings provide new insight into the functional effects of CMTDIC-associated *YARS1* variants and establish a tractable platform for investigating the cellular mechanisms underlying ARS-associated neuropathies. These findings also strengthen the argument for the use of tyrosine as an accessible and efficacious treatment for CMTDIC.

## 2. Results

Dominant pathogenic *YARS1* variants are associated with CMT disease, but the molecular and cellular impacts of these variants remain largely uncharacterized. We sought to determine the biochemical and cellular impacts of three YARS1 variants, G41R, D81I and E196Q, which are found in the N-terminal catalytic domain of the protein (Figure 1A).

### 2.1. CMT-Causative YARS1 Variants Impact Protein Thermal Stability

To determine the impact of YARS1 variants on the molecular level, we purified recombinant YARS1 wildtype, G41R, D81I and E196Q from *Escherichia coli*. Previous studies have shown that pathogenic YARS1 variants can alter protein thermal stability, specifically that G41R mildly increases thermal stability, and a similar E196K variant decreases stability [28]. To investigate the impacts of D81I and E196Q on the protein, we conducted a thermal stability assay, where protein unfolding is measured over a temperature range from 20 to 70 °C [22,23,29], and the melting temperature indicates the temperature at which half of the protein is unfolded. Here, the D81I and E196Q variants did not result in significantly altered thermal stability, while G41R led to a significant increase in thermal stability, by 4 °C from 51.9 °C to 56.1 °C, indicating increased rigidity of the recombinant protein (Figure 1B). We also measured the effects of ATP and tyrosine, on their own and in combination, on the recombinant wildtype and variant proteins. The wildtype protein displayed a significantly increased melting temperature for conditions where Tyr (54.3 °C) or both Tyr and ATP (54.7 °C) had been added (Figure 1B), which agrees with previously published data on the addition of Tyr to the wildtype protein. No change in the melting temperature was observed in any of the disease-causing proteins compared to the corresponding apo-protein upon substrate addition, indicating a loss of structural accommodation to the substrate-bound state.

Next, we investigated whether these structural changes were reflected in altered secondary structure. We first obtained circular dichroism (CD) spectra but found no measurable difference between wildtype proteins and variant proteins, either alone or in combination with the wildtype protein (Figure 1C,D). We therefore carried out limited tryptic digests at both 37 °C and 40 °C, where the purified recombinant protein is incubated with trypsin for increasing time periods to reveal subtle changes in the protein secondary structure [26,30]. The undigested proteins (C0 for starting material and C120 incubated for 120 min without trypsin) showed that all proteins are comparably stable over time (Figure 1E) at 37 °C. The wildtype protein showed a characteristic digestion pattern, where about half of the protein was cleaved after 30 min, with almost complete cleavage after 120 min at 37 °C (Figure 1E). In contrast, variants G41R and E196Q showed very few digestion products even after 120 min, indicating non-accessible cleavage sites (Figure 1E). The tryptic digest of D81I, on the other hand, is almost complete after 60 min at 37 °C, indicating a shift in the accessibility of tryptic digest sites (Figure 1E). At 40 °C, the digestion patterns for wildtype, G41R and E196Q are comparable to the reaction at 37 °C, but D81I showed a slightly slower degradation than at 37 °C. Overall, the thermal stability and tryptic digest data show that the variants cause an alteration in the structure of the protein.

### 2.2. CMT-DI Variants Do Not Impair Dimer Formation

**Figure 2:**
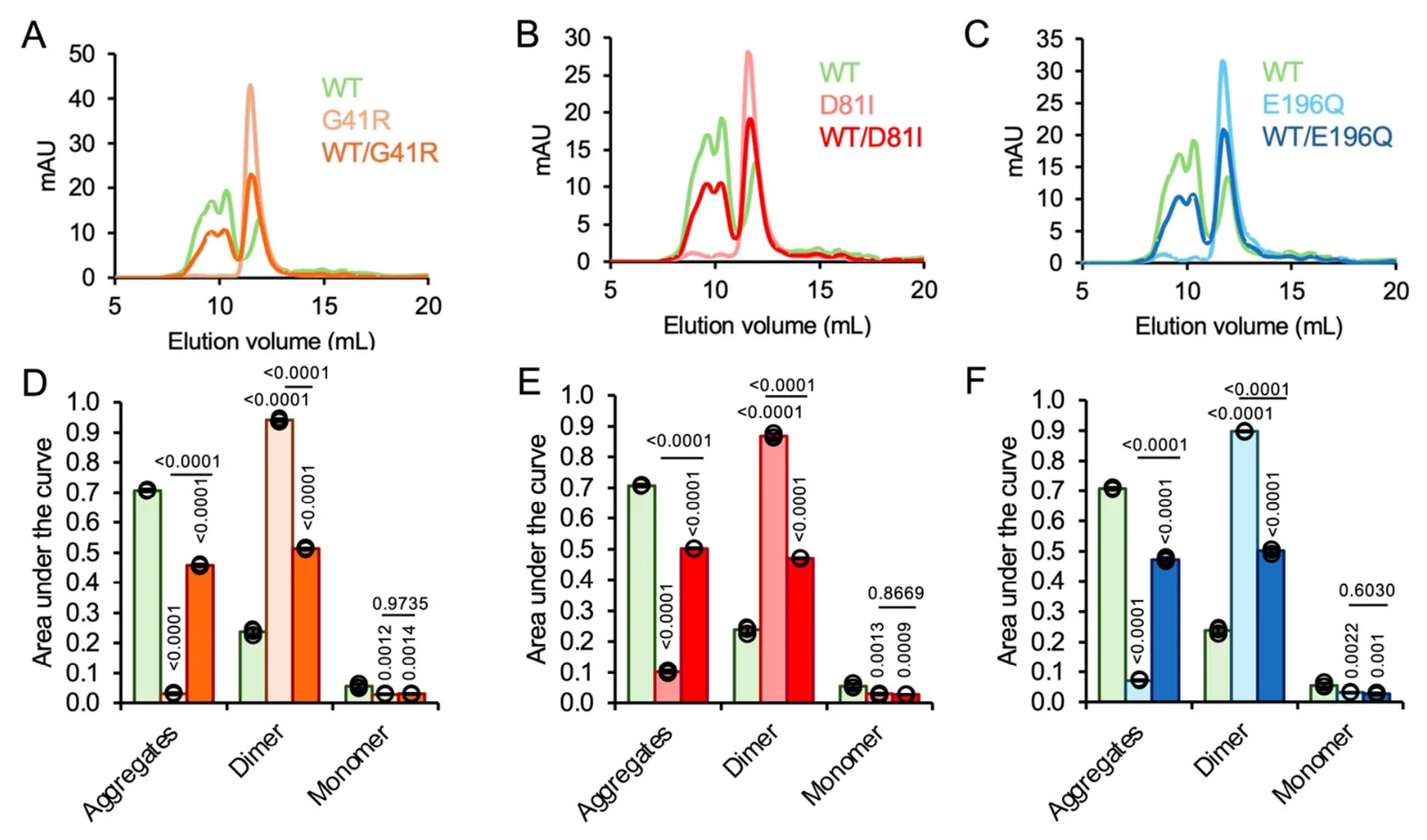
CMT-DI variants impact dimer formation. (**A**) Size-exclusion chromatography elution profiles of YARS1 WT, G41R, (**B**) D81I, and (**C**) E196Q and a 1:1 ratio of YARS1 WT and YARS1 variant recombinant proteins, and (**D**–**F**) quantification of area under the curve. In sum, 500 μL of 500 μg/mL recombinant protein was loaded on a Superdex™ 200 Increase 10/300 GL column from Cytiva and monitored by UV_280_. Statistics were determined using one-way ANOVA in GraphPad Prism. *p* values are indicated. *n* ≥ 3. Statistical comparisons to the WT are indicated directly above the error bars, which indicate one standard deviation.

Human YARS1 functions as a homodimer, and dimer disruption has been associated with disease [28]. We carried out size-exclusion chromatography to calculate the percentage of protein found forming dimers, determining the aggregate, dimer and monomer fractions for the wildtype and variant and the 1:1 combination of these proteins. The YARS1 wildtype eluted as 70% aggregate at 10 mL, 24% dimer at 12.5 mL and 6% monomer at around 15 mL (Figure 2A–F), as determined by a calibration curve generated with reference proteins of known molecular weights. Interestingly, all three variants exhibited less aggregation compared to the wildtype, where 87–94% of the variants on their own eluted as a dimer, with the aggregate fraction comprising only 3–10% of the total protein eluted (Figure 2A–F). When mixed in a 1:1 ratio with the wildtype protein, around 50% of the protein mixtures eluted as aggregates and 50% as dimers, with the monomer fraction remaining negligible. Overall, our data suggest that dimer formation is not disrupted in the YARS1 G41R, D81I, and E196Q CMTDIC variants; however, the dramatic reduction in aggregation in these variants could indicate altered surface hydrophobicity or conformational changes that affect oligomerization in vitro. All biochemical data is summarized in Table 1.

### 2.3. YARS1 Alleles Cause a Dominant Negative Phenotype in a Humanized Yeast Model

**Figure 3:**
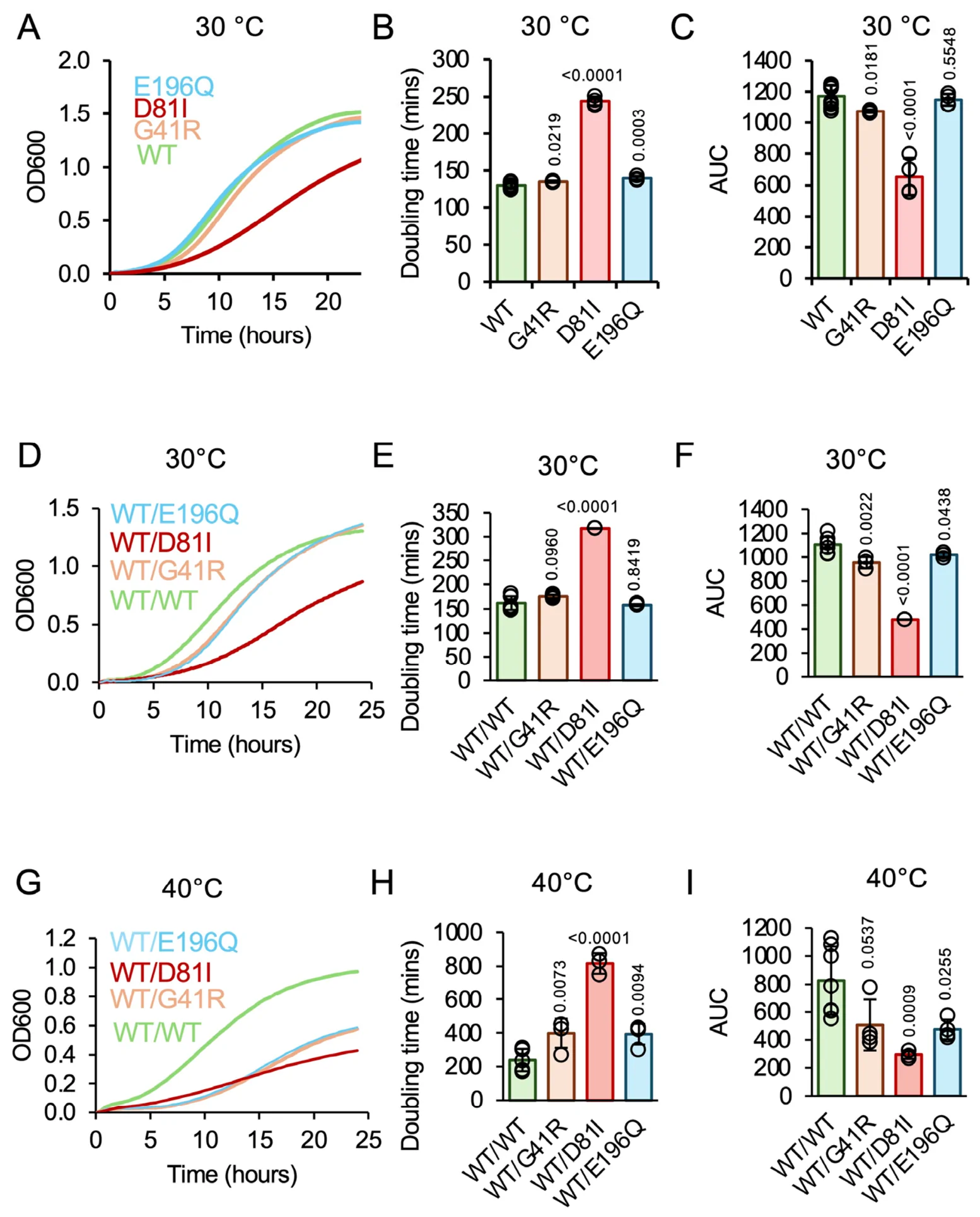
CMTDIC-associated YARS1 variants cause a growth defect in a humanized yeast model. Growth curves at 30 °C for YARS1 WT and CMTDIC-associated YARS1 variants, using a (**A**) monoallelic or (**D**) biallelic humanized yeast model. (**G**) Growth curves were also performed at 40 °C for the biallelic model. Yeast cultures were inoculated overnight in SD ura- leu- medium and normalized to an initial OD_600_ of 0.1 before incubation and shaking in a Synergy-H1 plate reader (BioTek, Winooski, VT, USA) for 24 h with 10 min read intervals. Doubling time at (**B**,**E**) 30 °C and (**H**) 40 °C. The doubling time was determined by calculating the slope of the exponential phase of the growth curve and using the following equation: doubling time = (log (2)/slope). Area under the curve (AUC) at (**C**,**F**) 30 °C and (**I**) 40 °C, reflecting total biomass accumulation. The area under the curve was determined using the ‘area under the curve’ analysis function in GraphPad Prism. Statistics were determined using one-way ANOVA in GraphPad Prism. *p* values compared to wildtype are indicated. Error bars represent one standard deviation. *n* ≥ 4.

Similar to earlier work with our previously generated humanized yeast HARS1 model [22,23,26,27], we generated a humanized yeast model for YARS1. In diploid yeast, one allele of the essential YARS1 is replaced by a kanamycin cassette, a plasmid-derived YARS1 is introduced, and yeast are sporulated and dissected, and haploid progeny analyzed. Haploid yeast resistant to kanamycin (no endogenous YARS1) rely on the plasmid-derived expression of wildtype human YARS1 or YARS1 variants. First, we tested whether the human wildtype *YARS1* and pathogenic variants can complement the yeast deletion strain. All three variants showed robust growth at 30 °C (Figure 3A). The doubling time of the wildtype human YARS1 (130 min) is within the range of a normal haploid yeast doubling time of around 120 min [22,31]. G41R (135 min) and E196Q (140 min) grew at slightly slower rates than the wildtype (WT) YARS1-expressing cells but showed no difference in area under the curve as a measure of total biomass accumulation (Figure 3B,C). D81I showed a more drastic increase in doubling time, by about 2-fold (244 min), and a 2-fold reduction in area under the curve (Figure 3B,C).

To recapitulate the dominant states of CMT variants, we generated model systems that express two human YARS1 variants from two separate vectors in the haploid yeast deletion background. To distinguish these models from yeast diploids, we termed these strains “biallelic expression” models. The yeast express both human wildtype YARS1 and the disease-causing variant from two separate plasmids, yielding strains with WT/WT, WT/G41R, WT/D81I or WT/E196Q genotypes. We obtained growth curves at 30 °C and quantified the doubling time and area under the curve (Figure 3D). The doubling times of the WT/WT-expressing strains were approximately 160 min, showing that a second copy of the wildtype allele affects doubling times compared to the strain with a single WT copy (Figure 3E), where increased YARS abundance slightly impacts the delicate balance of translation. At 30 °C, the doubling times of WT/G41R, and WT/E196Q resembled the doubling time of WT/WT, though the area under the curve as measurement of total biomass accumulation was significantly reduced by about 10% (Figure 3F). In contrast, WT/D81I was starkly reduced in growth, with a 2-fold increase in doubling time to 318 min, and a 2.3-fold reduction in biomass accumulation (Figure 3E,F). In the biallelic expression model all strains express a fully functional copy of the wildtype allele. If the variants would lead to a loss of function rather than a dominant negative mechanism, we would expect a reduction in the doubling time that is similar to the doubling times of the yeast that only expresses the wildtype allele from a single plasmid. These data show that all three variants reduce yeast viability in a dominant negative manner. All yeast growth data is summarized in Table 2 and Table 3.

### 2.4. Pathogenic YARS1 Phenotypes Are Exacerbated in Response to Heat Stress

Some ARS variants, such as HARS1 Y454S [22,32,33], show a reduction in activity and exacerbation of disease phenotypes during febrile episodes. We therefore tested whether growing the humanized yeast strains under increased temperatures would lead to more pronounced phenotypes in the humanized yeast. When grown at 40 °C, a temperature recapitulating human fever, the WT/WT strain showed a slightly increased doubling time of 234 min (Figure 3H). When grown at 40 °C, all three CMT variants showed significant increases in doubling time and decreases in area under the curve (Figure 3H,I). All strains remain viable, with WT/G41R (452 min) and WT/E196Q (301 min) showing a significant increase in doubling time. WT/D81I remains the strain with the most pronounced phenotype, with a 4-fold increase in doubling time (815 min). These data show that the three CMT-causative alleles lead to a dominant negative phenotype in yeast that is exacerbated under heat stress.

### 2.5. YARS1 D81I and G41R Lead to Proteomic Changes in a Humanized Yeast Model

**Figure 4:**
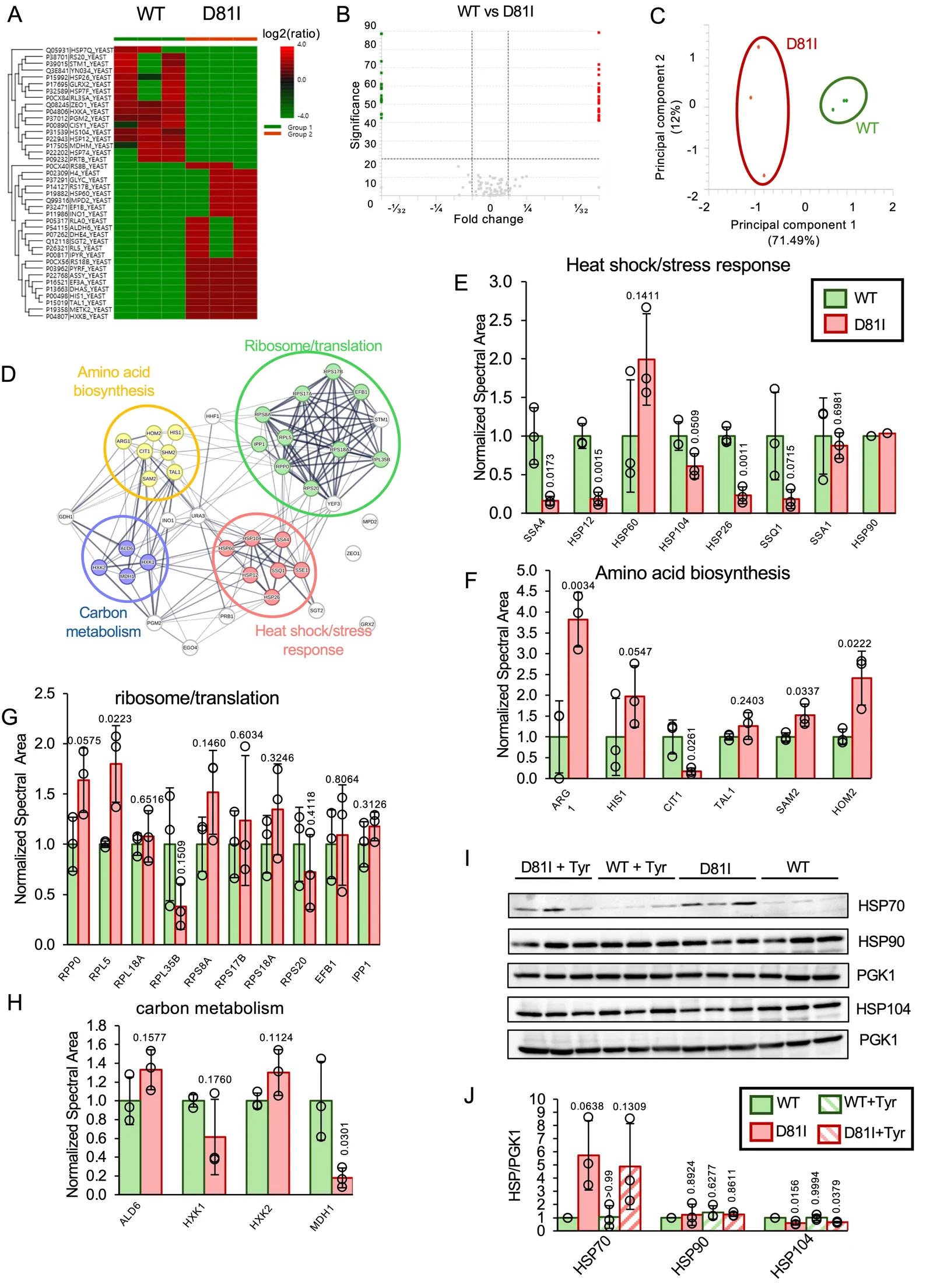
YARS1 D81I results in proteomic perturbations in a monoallelic humanized yeast model. (**A**) Heat map, (**B**) volcano plot and (**C**) principal component analysis of proteins significantly up- (red) or down- (green) regulated in YARS1 D81I-expressing cells compared to WT YARS1-expressing cells, by at least 2-fold. (**D**) Pathway functional enrichment analysis of proteins differentially regulated by at least 2-fold with at least one quantified peptide, generated with STRING. Bar graph of (**E**) heat shock; (**F**) amino acid biosynthesis; and (**G**) ribosomal or translational and (**H**) carbon metabolism proteins changed in abundance with at least three quantified peptides in D81I normalized to wildtype YARS1-expressing yeast. (**I**) Western blotting and (**J**) quantification of HSP70, 90 and 104 protein abundance in yeast lysates expressing YARS WT or D81I supplemented with or without tyrosine. Quantification of blots was performed using ImageJ (version 1.54). Statistics were determined using Student’s *t*-test in GraphPad Prism. *p* values are indicated. *n* = 3. α = 0.05. For HSP90, quantifiable peptides were detected in only two replicates of the wildtype samples, so no statistical analysis was carried out. Full protein names are listed in Data File S1.

We have previously shown that the expression of pathogenic variants in humanized yeast models causes a disruption of the proteome [22,26,27]. To assess whether the expression of variant YARS1 alters the proteome, we chose two of the variants, G41R and D81I. For D81I, we compared the proteome of the wildtype YARS1-expressing yeast to that of yeast relying on YARS1 D81I. We found that D81I caused a somewhat limited perturbation of the proteome, with a decrease in abundance in several chaperones, and an increase in proteins involved in amino acid metabolism (Figure 4A–F). For example, SSA4, one of the five heat shock protein 70 (HSP70) in *S. cerevisiae*, is ~10-fold downregulated in the D81I strain. Similarly, heat shock protein 12 (HSP12), one of the two major small heat shock proteins and heat shock protein 26 (HSP26), which functions in the recovery of misfolded proteins and prevents aggregation, are also ~10-fold reduced in abundance. General anti-stress chaperone heat shock protein 104 (HSP104) is downregulated by about 2-fold, which was validated by Western blotting, where HSP104 protein abundance was again shown to be reduced by nearly 2-fold (Figure 4E,I,J). Western blotting was also performed probing for the protein folding chaperone heat shock protein 90 (HSP90), which revealed no change in D81I yeast lysates compared to WT, confirming the proteomics data. For HSP70, we also found no difference in abundance in the Western blots. While the HSP70 antibody detects SSA4 (reduced in D81I in the proteomic data), it also detects other HSP70 family proteins. These epitopes are SSA1, SSA2, SSC1 (not changed in proteomics); SSA3, KAR2 (downregulated in proteomics data); and SSB2 (upregulated in proteomics data, see accession code). We therefore conclude that the HSP70 protein family is overall unchanged, though individual HSP70 protein members may be altered in abundance.

These data indicate that the response to protein aggregation and protein misfolding is not activated in D81I yeast. In contrast, several proteins in amino acid metabolism are increased in abundance; for example, the ARG1 encoding argininosuccinate synthase, which catalyzes the eighth step in arginine biosynthesis, is upregulated 3.8-fold. SAM2, a AdoMet synthetase generating S-AdenosylMethionine (AdoMet) from Methionine (Met) and ATP, is significantly upregulated by 1.5-fold, and HOM2, which encodes aspartate-beta-semialdehyde dehydrogenase, an enzyme involved in synthesizing threonine and methionine, is upregulated by 2.5-fold (Figure 4F). In contrast, CIT1, a mitochondrial citrate synthase involved in citrate metabolism and acetyl-CoA catabolism, is 5-fold downregulated. These data indicate a disruption of amino acid homeostasis in D81I yeast. While several ribosomal proteins are deregulated in D81I cells, only RPL5, a component of the hexameric 5S RNP precursor complex, is significantly upregulated (1.8-fold) when using a cut-off of at least 3 peptides for quantification (Figure 4G). Similarly, while several proteins involved in carbon metabolism are upregulated by at least 2-fold when quantifying 1 peptide (Figure 4D,H), only one protein is significantly upregulated when the 3-peptide minimum is applied. This protein, the Mitochondrial malate dehydrogenase (MDH1), is downregulated by 5-fold in D81I yeast (Figure 4H). Overall, these data show that the D81I proteome is mostly intact, with minor perturbation due to D81I expression, and these changes in the proteome are likely not causative for the pronounced phenotype of D81I-expressing cells.

**Figure 5:**
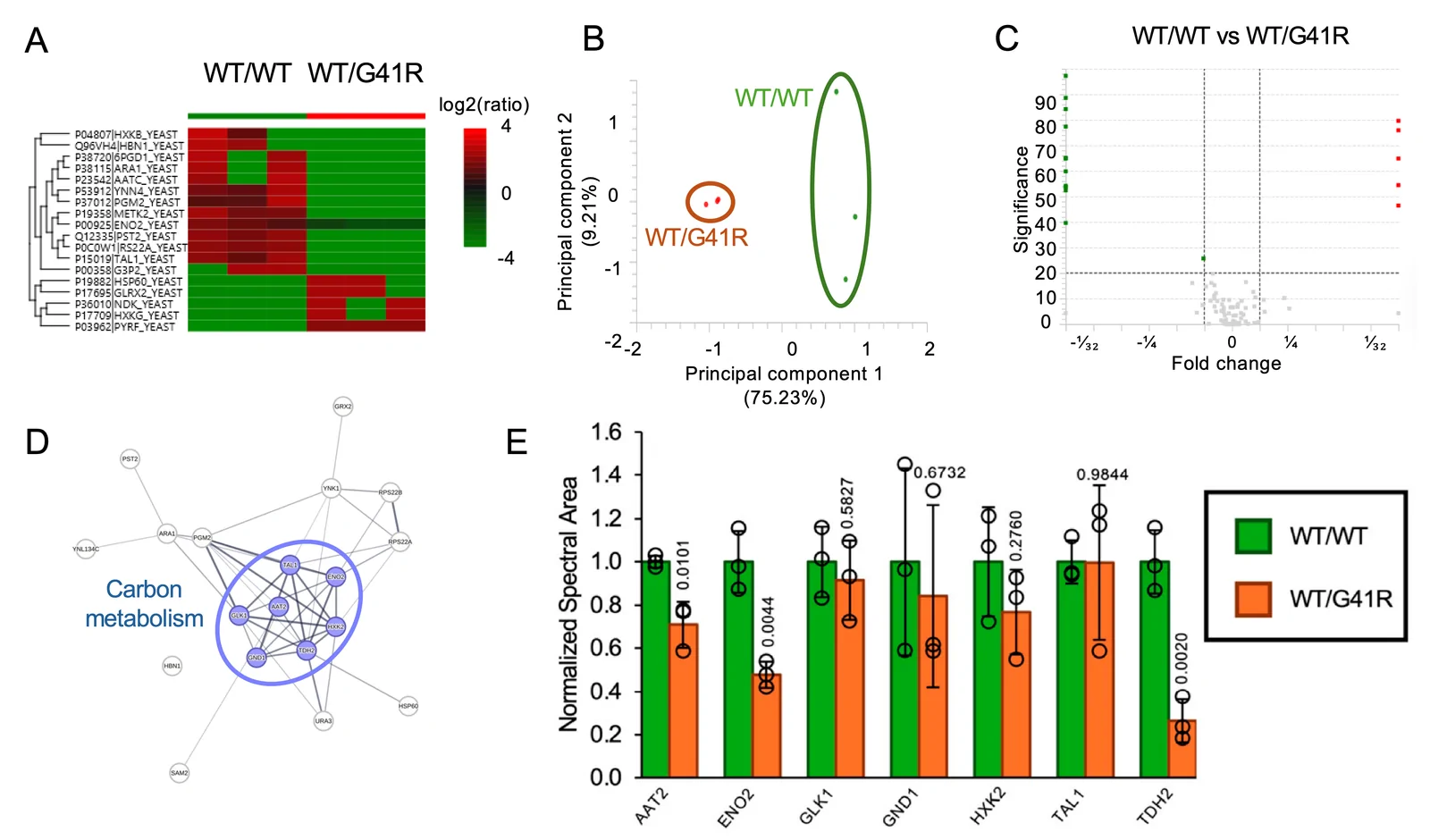
YARS1 G41R impacts carbon metabolism in a biallelic humanized yeast model. (**A**) Heat map, (**B**) volcano plot and (**C**) principal component analysis of proteins significantly up- (red) or down- (green) regulated in YARS1 G41R-expressing cells compared to WT YARS1-expressing cells, by at least 2-fold with at least one quantified peptide. (**D**) Pathway functional enrichment analysis of proteins differentially regulated by at least 2-fold, generated with STRING. (**E**) Bar graph of carbon metabolism proteins changed in abundance in WT/G41R normalized to WT/WT YARS1-expressing yeast with at least three quantified peptides. Statistics determined using Student’s *t*-test in GraphPad Prism. α = 0.05. *p* values compared to the corresponding WT value are indicated. Full protein names are listed in Data File S1.

While monoallelic yeast provides an excellent tool for exacerbating phenotypes, CMT-causative alleles in humans are accompanied by a wildtype allele. While the phenotype of WT/G41R-expressing yeast is relatively mild compared to that of WT/WT yeast, this allele combination nonetheless causes CMT. We therefore sought to test whether proteomic changes are found in these cells. As expected, the proteomic changes between WT/WT and WT/G41R cells are even less pronounced than the changes found in D81I yeast (Figure 5A–C). Interestingly, the most pronounced changes were found in carbon metabolism (Figure 5D,E). Aspartate aminotransferase (AAT2, 0.7-fold), phosphopyruvate hydratase (ENO2, 0.5-fold) and Glyceraldehyde-3-phosphate dehydrogenase (GAPDH), isozyme 2 (TDH2, 0.3-fold) were downregulated in the WT/G41R yeast, indicating a major reduction in glycolysis and metabolic activity (Figure 5E). Nonetheless, it appears that these changes, in addition to the proteomic adaptations in D81I and G41R cells, may be an accommodative reaction to slowed metabolic demands and protein synthesis.

### 2.6. YARS1 Alleles Do Not Cause Protein Aggregation or Detectable Mistranslation in a Humanized Yeast Model

**Figure 6:**
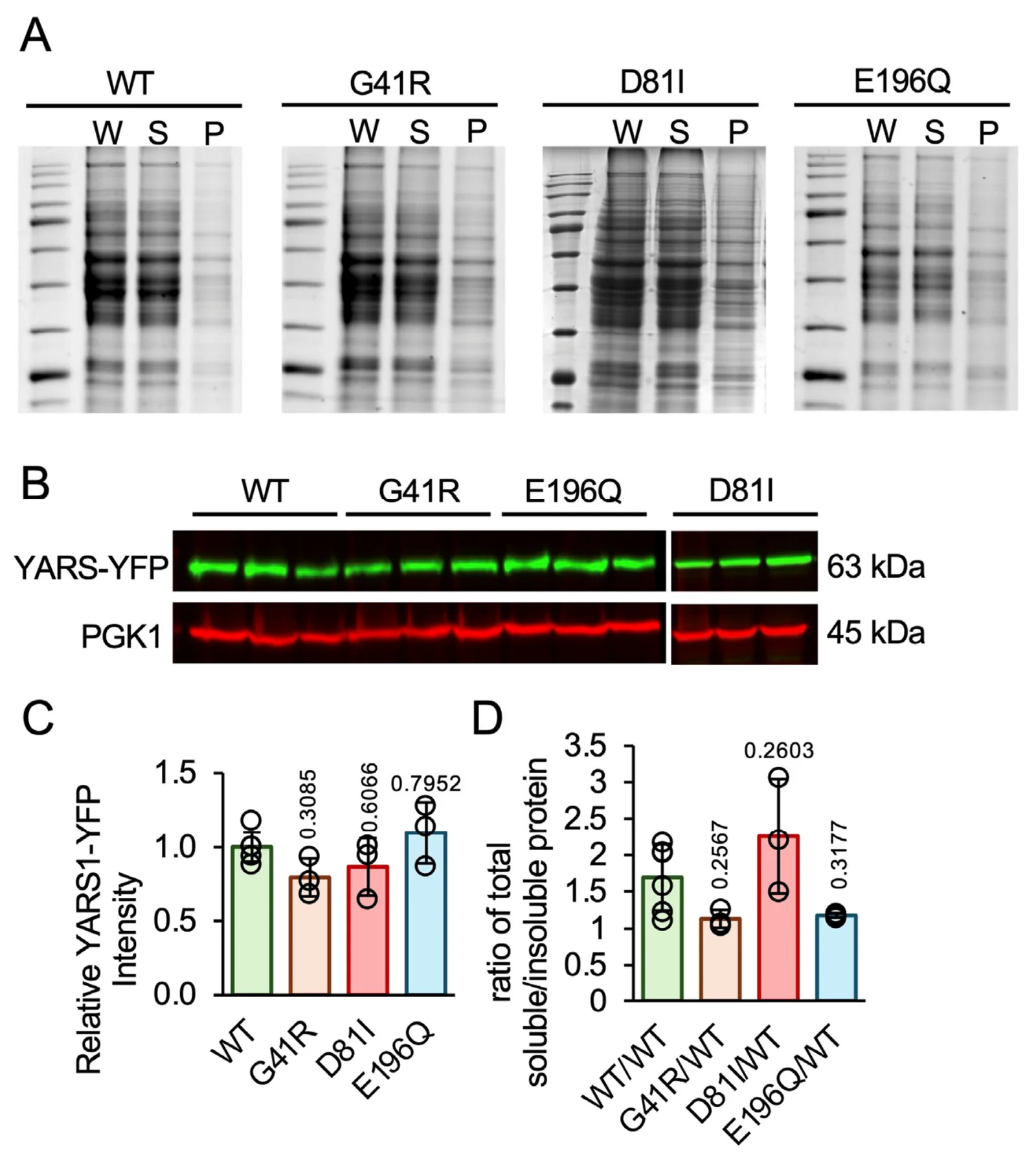
CMTDIC-associated YARS1 variants do not significantly impact protein abundance or global protein solubility in a humanized yeast model. (**A**) SDS gels of sedimentation assay for WT YARS1 and CMTDIC-associated YARS1 variants. Equal volumes of whole lysate (W), soluble fraction (S) and insoluble fractions (P) portions extracted from different yeast variants in the sedimentation assay were loaded on to 15% SDS gels. A ratio above one represents a higher proportion of soluble fraction compared to the insoluble fraction. (**B**) Western blot for YARS1 in the whole lysate fraction. An equal volume of whole lysate extracted from each yeast strain was loaded and blotted for YARS1. (**C**,**D**) Quantification of gels and blots was performed using ImageJ. (**C**) Quantification of blots in (**B**). (**D**) Ratio of soluble to insoluble total protein fraction for WT YARS1 and CMTDIC-associated YARS1 variants. Statistics were determined using one-way ANOVA in GraphPad Prism. *p* values compared to wildtype are indicated. *n* ≥ 3.

Our proteomic data is not indicative of global changes in the proteome. Nonetheless, proteomic analysis does not discriminate between properly folded and aggregated proteins. To assess whether D81I or G41R cause protein aggregation in yeast, we carried out a sedimentation assay. Here, soluble proteins are separated from insoluble proteins and separated by SDS gel to calculate the ratio of soluble and insoluble proteins [34]. We found no change in protein aggregation in any of the three variants (Figure 6A,D). In addition, we quantified the abundance of YARS1 by Western blotting and found no change in YARS1 abundance in any of the three variants compared to wildtype (Figure 6B,C). To probe for mistranslation events which had been elucidated previously in our humanized yeast model for HARS1 variants [22,26], SPIDER peptide mutation and homology searching analysis were performed in PEAKS. However, no mistranslation events were found to be above baseline in the proteome for YARS1 D81I or YARS1 WT/G41R yeast. These data indicate that reduced YARS1 abundance and protein aggregation are unlikely to contribute to the yeast phenotypes; however, YARS1-specific aggregation could still be occurring.

### 2.7. Dominant Negative Growth Phenotypes Are Rescued by Tyrosine Supplementation

**Figure 7:**
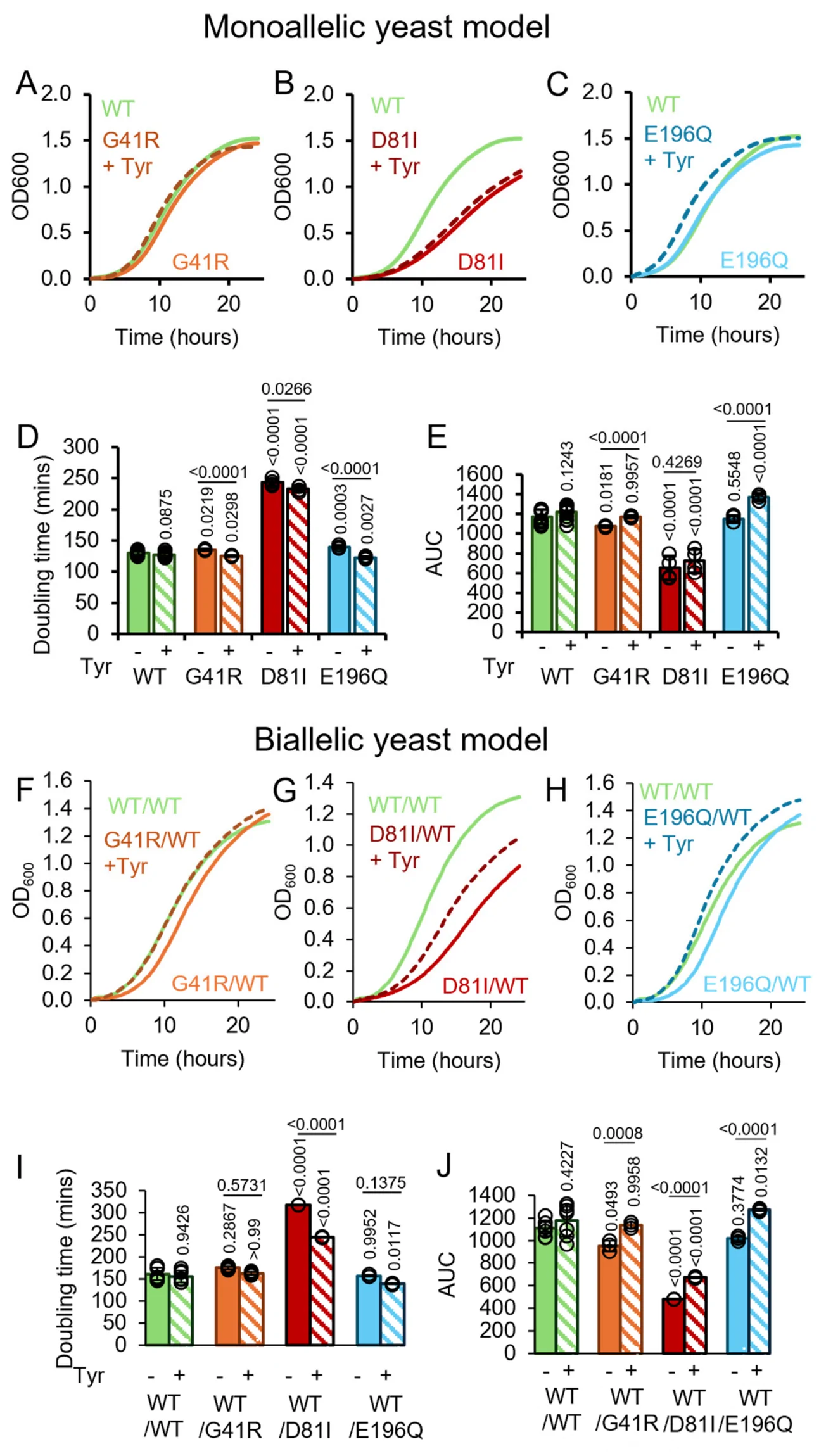
Tyrosine rescues a growth defect in a humanized yeast model of CMTDIC. Growth curves for YARS1 WT and CMTDIC-associated YARS1 variants at 30 °C. (**A**) G41R, (**B**) D81I and (**C**) E196Q supplemented with or without 200 mg/L tyrosine in a monoallelic humanized yeast model. Growth curves for YARS1 WT and CMTDIC-associated YARS1 variants (**F**) G41R, (**G**) D81I, and (**H**) E196Q supplemented with or without 200 mg/L tyrosine in a biallelic humanized yeast model at 30 °C. Quantifications of (**D**,**I**) doubling time and (**E**,**J**) area under the curve for growth curves at 30 °C. Dotted lines indicate tyrosine supplemented variants. Statistics were determined using one-way ANOVA in GraphPad Prism. *p* values are indicated. *n* ≥ 3. Statistical comparisons to the WT are indicated directly above the bars. Error bars represent one standard deviation.

In recent years, amino acid supplementation has been recognized as a potentially simple and powerful therapeutic intervention for recessive ARS alleles [17,22,23,25,35]. Indeed, some improvement was found for patients in patients with recessive biallelic and homozygous *YARS1* variants [25]. Nonetheless, scientific evidence that amino acid supplementation may be beneficial in dominant cases is scarce. We recently showed that some dominant *HARS1* alleles may indeed not benefit, where histidine supplementation led to increased mistranslation and protein aggregation in humanized yeast [22,26]. It is therefore imperative to test the impact of amino acid supplementation prior to therapeutic intervention. We therefore tested whether tyrosine supplementation could alleviate the yeast growth phenotypes. First, we tested whether tyrosine supplementation could rescue the growth phenotypes in the haploid model expressing only the disease alleles (Figure 7A–D). We supplemented at a concentration of 200 mg/L, which has been shown to be efficacious in producing a therapeutic effect in other dominant CMT-associated models [23] and is below amounts used with no side effects for other diseases [36]. We found that tyrosine did not alter the doubling times or area under the curve for the wildtype-expressing strain, indicating no negative effect on growth. Doubling times for all three pathogenic variants were reduced upon tyrosine supplementation, although the reduction itself was minimal (Figure 7A–D). Similarly, the area under the curve was significantly but minimally increased for all three strains (Figure 7E). To recapitulate the phenotypes found in humans, we used the dual plasmid model, testing growth parameters for WT/WT, WT/G41R, WT/D81I and WT/E196Q. Again, no difference in doubling time or area under the curve was observed for the WT/WT strain when supplemented with tyrosine (Figure 7I,J). As mentioned above, WT/G41R and WT/E196Q do not show a reduction in doubling time at 30 °C, and tyrosine supplementation did not increase or decrease doubling time (Figure 7F,H–J). Excitingly, the growth deficit for WT/D81I was partially restored upon tyrosine supplementation, where the doubling time was restored to 244 min (WT/WT is 160 min, WT/D81I is 318 min, Figure 7G,I). The area under the curve as a measurement of biomass accumulation was also partially restored for WT/D81I (Figure 7J). As outlined above, the area under the curve was significantly reduced for both WT/G41R and WT/E196Q, and excitingly, this measurement was completely restored for WT/G41R and WT/E196Q (Figure 7J). Interestingly, the tyrosine-supplemented WT/E196Q strain even showed slightly enhanced growth over the WT/WT strain, though this small ~5% increase may not be biologically significant (Figure 7H,J).

**Figure 8:**
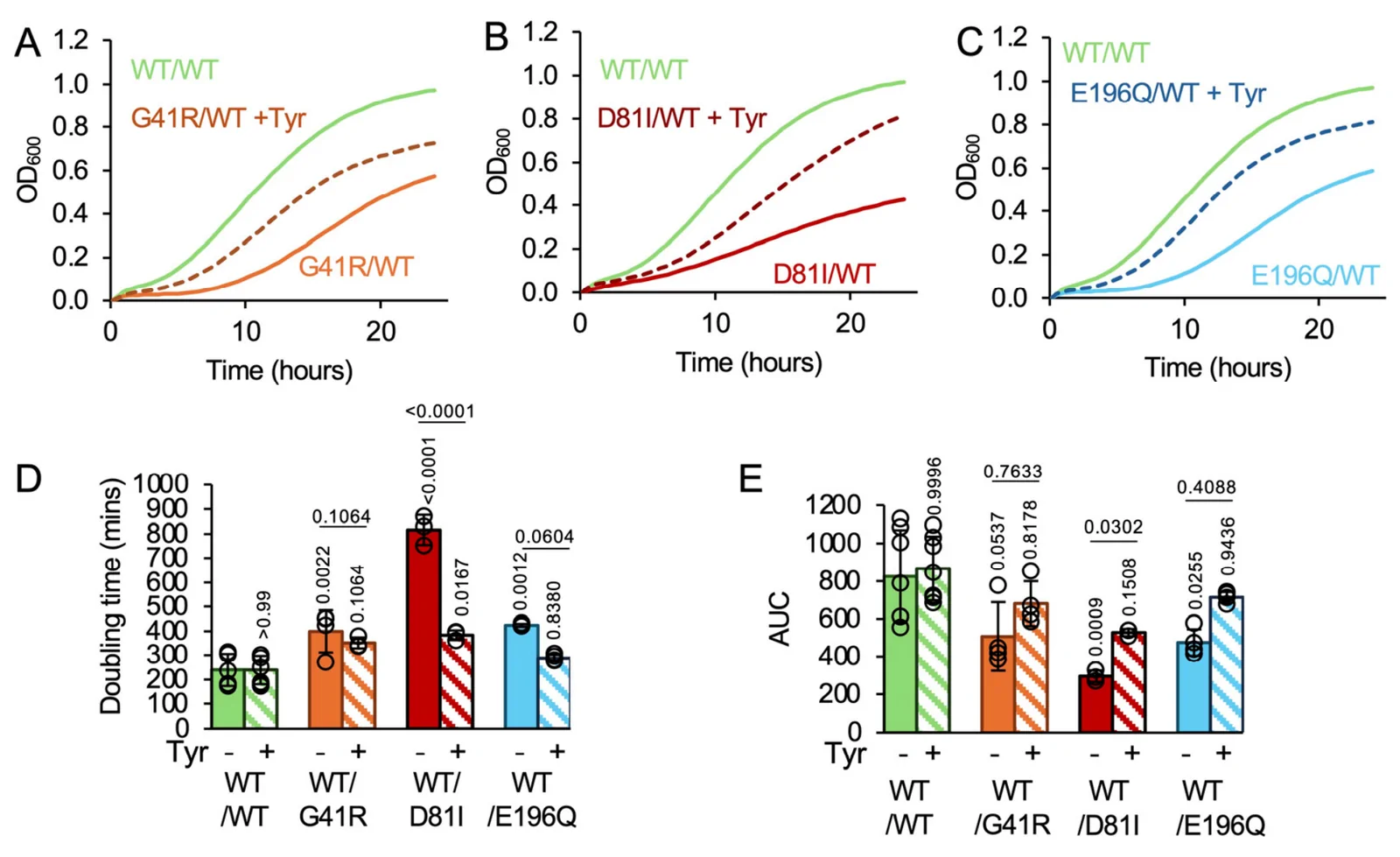
Tyrosine rescues a growth defect in a humanized yeast model of CMTDIC at 40 °C. Growth curves for YARS1 WT and CMTDIC-associated YARS1 variants at 40 °C. (**A**) WT/G41R, (**B**) WT/D81I and (**C**) WT/E196Q supplemented with or without 200 mg/L tyrosine in a monoallelic humanized yeast model. Growth curves for YARS1 WT and CMTDIC-associated YARS1 variants (**A**) G41R, (**B**) D81I, and (**C**) E196Q supplemented with or without 200 mg/L tyrosine in a biallelic humanized yeast model at 40 °C. Quantification of (**D**) doubling time and (**E**) area under the curve for growth curves at 40 °C. Dotted lines indicated tyrosine supplemented variants. Statistics were determined using one-way ANOVA in GraphPad Prism. *p* values are indicated. *n* ≥ 3. Statistical comparisons to the WT are indicated directly above the bars. Error bars represent one standard deviation.

Next, we tested whether the more pronounced phenotypes at 40 °C could also be rescued by tyrosine supplementation. All three yeast strains showed a pronounced rescue with tyrosine (Figure 8). The doubling times of tyrosine-supplemented strains for WT/G41R and WT/E196Q were no longer significantly different from the WT/WT-expressing yeast (Figure 8A,D). For WT/D81I, the doubling time was also significantly reduced, from 815 min to 383 min, yet remained statistically different from the WT/WT (239 min) strain (Figure 8B,D). As for the area under the curve, all tyrosine-supplemented strains showed significantly decreased values for unsupplemented strains, which were rescued to values no longer significantly different from the wildtype strains, indicating a complete rescue (Figure 8A–C,E). These data demonstrate that tyrosine supplementation can fully (G41R, E196Q) or partially (D81I) rescue the growth phenotypes in humanized yeast models.

## 3. Discussion

Disease-causing variants in *YARS1* can be either dominant or recessive. Dominant variants are associated with intermediate Charcot–Marie–Tooth disease (CMT-DI), and recessive disorders usually present as multisystem disorders. Recessive disorders are usually thought to be loss-of-function alleles, and many examples of bi-allelic [25,37,38] and homozygous disorders have been described in the literature [37,39,40,41]. While recessive *YARS1* alleles appear to be more common, only a few cases of dominant alleles have been described in the literature. The most-researched variants are YARS1 G41R, E196K, and 153–156 delVKQV [9], where biochemical analysis [10,28], mouse models [42,43,44] and Drosophila [10] models have been generated. We here investigated three CMT-associated variants in biochemical assays and yeast models. In previous studies, we found that dominant negative alleles in HARS1 can cause mistranslation and the activation of the integrated stress response [23,26,27], and that these toxic gain-of-function consequences can be captured in yeast models, Interestingly, we found mistranslation above background levels in the humanized YARS1 yeast models, and only very limited activation of the heat shock response, as well as no increase in the accumulation of unfolded protein. The impact of the pathogenic YARS variants on the yeast proteome was relatively minor, at least compared to major rearrangement of the proteome [23,26,27] and mistranslation events [45] found in humanized yeast models and in human cell models for NARS disease. We therefore deduce that the dominant alleles identified in our studies may decrease the amount of catalytically competent dimers, as previously shown for dominant negative NARS1 alleles [45]. While all wildtype/variant mixtures formed dimers, as evidenced by the increased dimer formation or the recombinant proteins, it is likely that these dimers may not be as catalytically competent as wildtype homodimers, since the variants all cause changes to the protein structure, as detected by thermal stability and tryptic digest assays. Catalytic (in)competence may thus be the disease-causing factor, rather than toxic mistranslation.

### 3.1. YARS1 Variants Cause Distinct Protein Changes and Phenotypes in a Humanized Yeast Model

YARS1 G41 is a highly conserved residue involved in the formation of tyrosyl-adenylate, and the pathogenic G41R variant was first reported in a study that also identified two other disease-causing variants, E196K and 153–156 delVKQV [9]. That study found that both G41R and E196K reduced activity in a pyrophosphate release assay, showing impairment in the first step of amino acid activation [9]. A subsequent study found that G41R caused an almost complete loss of aminoacylation, and the 153–156 delVKQV variant had significantly decreased activity, while E196K retained full aminoacylation activity [10]. Our data on G41R agrees with a previous study finding that this variant does not obscure dimerization of the YARS1 protein [28], but leads to structural alteration, as demonstrated by decreased susceptibility to a limited tryptic digest. When mapped onto the yeast Tys1 protein, E196K showed reduced growth, and no complementation was observed for G41R [9,28]. For the human protein, we show that YARS1 G41R must retain some enzymatic activity, since human G41R complemented a yeast knockout in the absence of a wildtype *YARS1* allele. Interestingly, despite a previously suggested loss of function in G41R, this variant displayed a dominant negative phenotype, where the growth phenotype persists in a biallelic expression model with a fully functional human YARS1 expressed alongside the G41R variant.

Interestingly, the well-studied E196K variant is substantially different from the E196Q variant investigated here. E196 is predicted to interact with tRNA^Tyr^, and is associated with hereditary neuropathy, and both E196K and E196Q were previously shown to only partially complement a yeast deletion strain at 37 °C [12]. Similarly, we observed a small growth deficit at 30 °C and a more pronounced phenotype at 40 °C, one which persists in a heterozygous strain, indicating a dominant negative mechanism. On the protein level, and in contrast to E196K, E196Q does not appear to impair dimerization, while E196K showed a marked decrease in a previous study [28]. This is likely due to the different nature of the substitute amino acid, where a replacement of negatively charged glutamate by a larger and positively charged lysine disrupts dimer formation, but a polar, uncharged amino acid of similar size, like glutamine, may be compatible with dimerization. Nonetheless, we found that the structure of E196Q is likely impaired, as demonstrated by decreased thermal stability and reduced susceptibility to tryptic digest, which may contribute to reduced translational fidelity in yeast.

Similar to the YARS1 E196Q, very little data is available on the more recently discovered YARS1 D81I variant, which is caused by a di-nucleotide substitution in *YARS1*, c.241_242GA>AT, identified in a late-onset CMT patient with intermediate type CMT [13]. A functional analysis showed that this variant causes axon degeneration and cell death as well as reduced protein synthesis when overexpressed in primary mouse sensory neurons [46]. We found that D81I elicits a strong phenotype in humanized yeast, with a drastic reduction in viability. The D81I protein is highly susceptible to limited tryptic digest, indicating a change in structural accessibility and conformational state. While the growth phenotype of D81I variants is more pronounced than those of G41R and E196Q, the cells remain viable when only this variant is expressed, indicating sufficient aminoacylation activity. It should also be noted that in agreement with the dominant character of the variant, the dominant negative phenotype is also recapitulated in the yeast model. Overall, these data demonstrate that the dominant negative mechanism of the CMT-causative alleles can be captured in our yeast models.

### 3.2. Amino Acid Supplementation in ARS Diseases

While no known cure is available for any ARS-related disease to date, recent approaches aiming to ameliorate symptoms have focused on amino acid supplementation for recessive disorders. Increased substrate availability can compensate for weak residual protein activity in biallelic/recessive disorders [47], or can support the remaining competent wildtype heterodimers in dominant alleles [48]. Amino acid supplementation can also stabilize the recombinant variant protein [27,49], though this was not the case in the YARS variants investigated here. Tyrosine, however, is able to stabilize the WT YARS1 homodimer, as reflected in our thermal stability assay, and subsequently may have a stabilizing effect for heterodimers comprised of WT and variant YARS1 protein subunits. Fascinatingly, a recent study in *Caenorhabditis elegans* showed that siRNA-mediated depletion of all mitochondrial synthetases in *C. elegans* can be rescued by amino acid supplementation [50]. In patients, tyrosine supplementation showed some benefit in children with recessive *YARS1* deficiency [25,37], and we recently showed in a comprehensive clinical trial that children with a homozygous HARS variant treated with histidine showed marked improvement, and supplementation prevented hearing and vision deterioration. Similarly, patients with several other recessive ARS disorders were treated with cognate amino acids, with results ranging from encouraging [17] to dramatically positive [24,51,52]. Data on amino acid supplementation in dominant disorders remains scarce. We recently showed that histidine supplementation in humanized models for CMT can be beneficial in some cases but has no effect or even negatively impacts humanized yeast growth in other cases. It is therefore imperative to test amino acid supplementation in vitro prior to therapeutic intervention in patients [2,53,54]. Nonetheless, these data for histidyl-tRNA synthetase and our recent study in patient-derived cells for dominant negative variants in asparaginyl-tRNA synthetase show that cognate amino acid supplementation can rescue defects in humanized yeast models, as well as cell models and patient-derived cells. Excitingly, our data shows that in humanized yeast models, tyrosine supplementation shows a clear rescue of the growth defect, indicating a favorable outcome for tyrosine supplementation. Notably, tyrosine stabilized the wildtype recombinant protein but does not have an impact on variant recombinant protein stability, indicating that protein stabilization by substrate binding is not a contributing factor to the rescue. Since the strains expressing only the pathogenic variant showed a minimal increase in growth, it is likely that the rescue is due to an improved function of the wildtype allele, though direct assays to measure aminoacylation would be required to determine the molecular mechanism of the rescue. In healthy adults, normal plasma tyrosine levels range from 30 to 120 μmol/L, with levels above 200 μmol/L considered high [55]. Concentrations used in our yeast growth assays are close to 1 mM, which exceeds healthy tyrosine plasma levels. Yeast, as a fast-dividing organism, may require higher tyrosine levels than may be required for human cells, and our data give an indication that tyrosine restored growth in YARS1 disease models. Nonetheless, extensive monitoring is required in patients, with side effects such as tyrosinemia and hypertension having been previously observed with tyrosine supplementation [25]. In conclusion, our data indicate that tyrosine supplementation, which has already been used in clinical studies for recessive *YARS1* disorders, may be beneficial to patients with dominant *YARS1* G41R, D81I or E196Q variants.

## 4. Materials and Methods

### 4.1. Generation of the Humanized YARS1 Yeast Model

Cloned human *YARS1* was transformed into *TYS1/tys1Δ* heterozygous deletion strain, *MATa*/*MATα*, *his3Δ1*/*his3Δ1*, *leu2Δ0*/*leu2Δ0*, *LYS2*/*lys2Δ0*, *MET15*/*met15Δ0*, *TYS1*/*tys1Δ::KanMX4*, and *ura3Δ0*/*ura3Δ0* (Horizon Discovery, Cambridge, UK). Transformants were selected on SC-Ura and individual transformants streaked onto YPD+G418. Individual colonies were inoculated into YPD liquid media and grown at 30° overnight, with shaking at 300 rpm. Overnight cultures were inoculated onto solid minimal sporulation media (1% potassium acetate, 5 mg/L histidine, 25 mg/L leucine, 7.5 mg/L lysine, and 5 mg/L uracil) and incubated at 25° for 3–5 days. Sporulation was monitored by microscopy and subjected to dissection. Dissected tetrads were replica plated onto YPD, YPD+G418, and SC-Ura to identify *tys1Δ::KanMX4* haploids containing plasmid-borne *YARS1*.

### 4.2. Yeast Plasmids and Growth Conditions

QuickChange site-directed mutagenesis methods were used to generate human *YARS1* variants in a donor vector, and all expression constructs were created via Gateway cloning. Yeast episomal plasmids p426-ccdB-GPD (URA3 plasmid) and p425-ccdB-GPD (LEU2 plasmid) were used to express human YARS1 as a YARS1-YFP fusion in *tys1Δ* yeast. Yeast cells were grown on yeast extract–peptone–dextrose (YPD) (10 g/L yeast extract, 20 g/L peptone, and 20 g/L dextrose) or selective synthetic defined (SD) medium (6.7 g/L yeast nitrogen base, 2% glucose, 60 mg/L L-isoleucine, 20 mg/L L-arginine, 40 mg/L L-lysine, 60 mg/L L-phenylalanine, 10 mg/L L-threonine, 10 mg/L L-methionine, and 10 mg/L adenine), supplemented with 20 g/L agar for solid medium growth. In sum, 60 mg/L L-leucine or 20 mg/L uracil were added to the SD medium as needed, depending on the selectivity marker. In the biallelic expression model, human wildtype YARS1 and a variant are co-expressed from two different plasmids. The allele-specific single-plasmid model was generated via plasmid shuffling, where yeast harboring both p426 and p425 plasmids were patched onto 5-fluoroorotic acid (5-foa) solid media to counter-select for the URA3 plasmid, generating yeast strains containing only the LEU2 plasmid expressing the YARS1 variant. For liquid growth curves, yeast cultures were grown overnight, normalized to OD_600_ = 0.1, and grown in SD medium in 96-well plates for 24 h. For supplementation with L-tyrosine, tyrosine was added to SD medium to a final concentration of 200 mg/L before plating. All growth curves were done with three biological replicates per condition and three technical replicates per biological. All yeast strains were grown at 30 °C, and growth curves were conducted at 30 °C and 40 °C.

### 4.3. Recombinant YARS1 Protein Purification

Human *YARS1* was cloned into a pDEST527 vector to produce a 6x-His tag expression construct and transformed into *Escherichia coli* BL21 Codon Plus. Precultures were grown overnight in LB medium containing 100 μg/mL ampicillin and 34 μg/mL chloramphenicol. The overnight culture was diluted 1:100 in LB medium with antibiotics and grown at 37 °C until OD_600_ = 0.6. Isopropyl β-D-thiogalactopyranoside (IPTG) was added to a final concentration of 400 μM and the cells were left to grow for six hours at 37 °C. Cells were collected by centrifugation at 5000× *g* for 10 min. The pellet was resuspended in lysis buffer (50 mM NaH_2_PO_4_, 300 mM NaCl, 10 mM imidazole, and 1 mM phenyl-methyl-sulphonyl-fluoride (PMSF), pH 8.0) and lysed by sonication. The pellet and supernatant were separated by centrifugation at 35,000 rpm for 45 min. YARS1 proteins were purified by Ni-NTA affinity chromatography using HisPur^TM^ Ni-NTA resin (ThermoFisher Scientific, Waltham, MA, USA). Purification was carried out at 4 °C or on ice. Protein was dialyzed into 1× Buffer A (50 mM Tris-HCl, 300 mM NaCl, and 20 mM MgCl_2_, pH 8.0) with 1 mM dithiothreitol (DTT).

### 4.4. Thermostability and Dimerization Assays

Thermal shift assays were used to assess the thermal stability of YARS1 variants by measuring fluorescence intensity as protein unfolding occurred. In a 96-well 0.1 mL microplate, 2.5 μL of diluted 8X Protein Thermal Shift™ Dye and 5 μL of Protein Thermal Shift™ Buffer from the Protein Thermal Shift™ Dye Kit (ThermoFisher, 4461146) were combined with 12.5 μL of protein to a final concentration of 1 μM per well. Samples were also incubated with either 200 μM tyrosine or 100 μM ATP or both, all plated in quadruplicate. The plate was sealed and heated from 25 to 96 °C in increments of 1 °C in a QuantStudio™ 3 Real-Time PCR System (ThermoFisher Scientific), with fluorescence intensity measured every 0.049 °C. Using QuantStudio™ Design & Analysis Software v1.5.1, fluorescence was plotted against temperature, and data were fitted to the Boltzmann equation to estimate the melting temperature (T_m_). Size-exclusion chromatography was performed using the Superdex™ 200 Increase 10/300 GL column (Cytiva, 28990944, Marlborough, MA, USA) to assess the dimerization capabilities of YARS1 variants. The column was equilibrated in 1× Buffer A and 500 μL of 500 μg/mL protein was loaded onto the column for each run, for a total of three runs per sample. Elution of the protein was monitored by UV absorption at 280 nm.

### 4.5. CD Spectroscopy and Limited Tryptic Digest

For circular dichroism analysis, recombinant YARS1 protein was dialyzed into a UV-transparent CD buffer (10 mM potassium phosphate, 50 mM Na_2_SO_4_). CD spectra were obtained at a concentration of 500 μg/mL in a 0.1 cm cuvette using a Jasco J-1500 CD spectrometer (JASCO Corporation, Tokyo, Japan). A total of 3 spectra per sample were collected over the 260 to 180 nm range with a bandwidth of 1 nm at 4 °C, and the buffer spectrum was subtracted for baseline correction. For partial tryptic digestion, sequencing-grade modified trypsin (Promega, Madison, WI, USA) was prepared according to the manufacturer’s instructions. A quantity of 500 μg/mL of YARS1 protein was equilibrated in 1× Buffer A and incubated with trypsin in a 1000:1 ratio at 37 °C for 5, 30, 60, and 120 min. Controls were incubated without trypsin for 0 or 120 min. The reactions were quenched with 3X SDS dye (6% sodium-dodecyl-sulfate (SDS), 30% glycerol, 0.006% bromophenol blue, and 125 mM Tris-HCl (pH 6.8)), separated by 12% SDS polyacrylamide gel electrophoresis (SDS-PAGE), and stained with Coomassie blue.

### 4.6. Sedimentation Assay and Western Blotting

Sedimentation assays were adapted from Shiber et al. [34] Yeast cultures were normalized to OD_600_ = 1 and lysed (100 mM Tris-HCl (pH 7.5), 200 mM NaCl, 1 mM ethylenediaminetetraacetic acid (EDTA), 1 mM DTT, 5% glycerol, 0.5% Triton-X, 50 mM N-ethylmaleimide (NEM), 2 mM PMSF, and 3.2 mg/mL protease inhibitor); this was followed by manual glass bead disruption. Cell lysates were separated into the whole lysate, supernatant (soluble protein fraction), and pellet (insoluble protein fraction), combined with SUMEB buffer (8 M urea, 1% SDS, 10 mM MOPS, 10 mM EDTA, and 0.01% bromophenol blue), and then loaded onto a 12% SDS gel and stained with Coomassie blue. To determine the abundance of human YARS1 proteins in the yeast, 3X SDS dye was added to the whole lysate instead of SUMEB buffer, after which the samples were separated by 12% SDS-PAGE and transferred to a nitrocellulose membrane. Anti-GFP antibody (Abcam, ab32146, Cambridge, UK) was used at a ratio of 1:1000 to detect the YARS1-YFP fusion protein. Anti-HSP70 (Invitrogen, C92F3A-5, Waltham, MA, USA), anti-HSP90 (Invitrogen, MBH90AB), and anti-HSP104 (Abcam, ab69549) antibodies were used at a 1:1000 dilution to detect relevant heat shock proteins identified in the proteomics dataset. An antibody for PGK1 (Cell signaling, 68540, Danvers, MA, USA) was also used at a 1:1000 dilution as a loading control. Secondary antibodies IRDye^®^ 800CW, Donkey anti-Rabbit IgG and 680RD Goat anti-Mouse IgG (LI-COR, Lincoln, NE, USA, 926-32213 and 926-68070, respectively) were used at a ratio of 1:5000 and detected using the ChemiDoc MP Imaging system from Bio-Rad (Hercules, CA, USA).

### 4.7. Mass Spectrometry

Yeast cell pellets were resuspended in Y-PER (ThermoFisher Scientific) with Halt™ Protease and Phosphatase Inhibitor Cocktail (ThermoFisher Scientific). Each sample was sonicated at an amplitude of 60% for 5 min with cycles of 30 s on/30 off. Cell debris was pellet, and soluble protein was quantified by using a Pierce™ BCA Protein Assay kit (ThermoFisher Scientific, Waltham, MA, USA). An aliquot of 10 µg per sample was reduced with 100 mM tris(2-carboxyethyl)phosphine (TCEP) and alkylated with 200 mM IAA. The lysate was then digested with trypsin (New England Biolabs, Ipswich, MA, USA) at a 50:1 protein:enzyme ratio according to the manufacturer’s recommendations. Digested peptides were desalted by C18 clean-up, lyophilized, and stored until analysis.

The digested peptides were resuspended in Buffer A (0.1% FA) and injected into the timsTOF Pro (Bruker Daltonics, Bremen, Germany) by nanoflow liquid chromatography using a Thermo Scientific EASY-nLC 1000 (ThermoFisher, MA, USA). Liquid chromatography was performed using a constant flow of 300 µL/min and a 15 cm reversed-phase column with a 75 µm inner diameter filled with Reprosil C18 (PepSEP, Bruker, Germany). Mobile phase A was 0.1% formic acid and Mobile phase B was 99.9% acetonitrile, 0.1% formic acid. Peptide separation was carried out over 55.5 min as follows: linearly 4% A to 35% B over 50 min, an increase to 100% B over 30 s, and then held constant for 5 min to clean the column. Column equilibration was done automatically prior to sample loading.

The timsTOF Pro was outfitted with a Captivespray source (Bruker Daltonics, Bremen, Germany), operated in PASEF mode. Trapped ion mobility separation was achieved by using an accumulation time of 100 ms in the first TIMS region and ramps of the TIMS region from 0.85 V·s/cm^2^ to 1.30 V·s/cm^2^, with each ramp lasting 100 ms. MS and MS/MS scans were limited to 100 m/z to 1700 m/z, and a polygon filter was applied to the m/z and ion mobility dimensions to select for multiple charged ions most likely to be peptide precursors. Collision energy was applied as a function of ion mobility with a linear regression using the following parameter settings: 0.85 V·s/cm^2^ 27 eV, 1.30 V·s/cm^2^ 45 eV. TIMS voltage was calibrated using ions from the Agilent Tune Mix (*m*/*z* 622, 922, 1222). Active Exclusion of MS/MS scans were enabled at a setting of 0.40 min. Quadrupole isolation was set to 2 *m*/*z* for *m*/*z* less than 700, and 3.0 *m*/*z* for ions with an *m*/*z* greater than 800. A linear regression calculation was done automatically for ions in between *m*/*z* 700 and 800.

The raw data was analyzed in PEAKS Studio version 12.5. The data was searched against the *Saccharomyces cerevisiae* proteome database. Precursor ion mass error tolerance was set to 10 ppm and fragment ion mass error tolerance was set to 0.02 Da. Semi-specific cleavage with trypsin was selected with a maximum of 2 missed cleavages. A fixed modification of carbamidomethylation (+57.02 Da) on cysteine residues and variable modifications of N-terminal acetylation (+42.01 Da) and deamidation (+0.98 Da) on asparagine and glutamine, as well as oxidation (+15.99 Da) on methionine, were specified. A 1% false discovery rate (FDR) was set for the database search. A PSM score threshold of −10LgP > 20 was set for peptides and a protein score of −10LgP > 15.

### 4.8. Statistical Analysis

All statistical significance was determined by *t*-tests comparing the means and standard deviations of the control data and experimental data sets, or a one-way ANOVA test for multiple comparisons and corrected with Bonferroni correction or Tukey’s HSD test, as indicated. Statistical significance levels are annotated using asterisks (**** *p* < 0.0001, *** *p* < 0.001, ** *p* < 0.01, * *p* < 0.05, ns = not significant).

## Figures and Tables

**Table 1:** Biochemical results of YARS1 G41R, D81I, and E196Q variants compared to the wild-type YARS1. “+” indicates an increase, “−” indicates a decrease, and “+/−” indicates no difference in the qualitative or quantitative results being compared. For melting temperature, the variants in apo form are compared to the YARS1 WT reference and the substrate-supplemented conditions are compared to their apo forms. For tryptic digestion susceptibility, the 40 °C comparisons are relative to their 37 °C counterparts.

Variant	Melting Temperature (T_m_)				Tryptic Digestion Susceptibility		Dimerization
	Apoprotein	+ATP	+Tyr	+ATP, Tyr	37 °C	40 °C	
YARS1 WT	Reference	+/−	+	+	Reference	+/−	More aggregates than dimers, very small quantity of monomers
G41R	+	+/−	+/−	+/−	−	+/−	Decreased aggregates, increased dimers, similar quantity of monomers as YARS1 WT
D81I	+/−	+/−	+/−	+/−	+	+/−	
E196Q	−	+/−	+/−	+/−	−	+/−	

**Table 2:** Growth phenotypes of YARS1 G41R, D81I, and E196Q variants in a humanized yeast model of disease, compared to the wild-type YARS1 at 30 °C. “+” indicates an increase, “−” indicates a decrease, and “+/−” indicates no difference in the values being compared. All doubling time and area under the curve comparisons are relative to the no-tyrosine YARS1 WT or WT/WT reference.

Variant	Doubling Time (min)		Area Under the Curve	
	No Tyr	High Tyr	No Tyr	High Tyr
YARS1 WT	Reference	+/−	Reference	+/−
G41R	+	−	−	+/−
D81I	+	+	−	−
E196Q	+	−	+/−	+
WT/WT	Reference	+/−	Reference	+/−
WT/G41R	+/−	+/−	−	+/−
WT/D81I	+	+	−	−
WT/E196Q	+/−	−	+/−	+

**Table 3:** Growth phenotypes of YARS1 G41R, D81I, and E196Q variants in a humanized yeast model of disease, compared to the wild-type YARS1 at 40 °C. “+” indicates an increase, “−” indicates a decrease, and “+/−” indicates no difference in the values being compared. All doubling time and area under the curve comparisons are relative to the no-tyrosine WT/WT reference.

Variant	Doubling Time (min)		Area Under the Curve	
	No Tyr	High Tyr	No Tyr	High Tyr
WT/WT	Reference	+/−	Reference	+/−
WT/G41R	+	+/−	−	+/−
WT/D81I	+	+	−	+/−
WT/E196Q	+/−	−	−	+/−

## Data Availability

The data is available in a publicly accessible repository. The mass spectrometry proteomics data have been deposited in the ProteomeXchange Consortium via the PRIDE partner repository with the dataset identifier PXD078702.

## Supplementary Materials

The following supporting information can be downloaded at: https://www.mdpi.com/article/10.3390/ijms27167481/s1.
